# Surgical Simulation of a Posttraumatic Spinal Cord Glial Scar in Rats

**DOI:** 10.32607/20758251-2019-11-3-75-81

**Published:** 2019

**Authors:** G. B. Telegin, A. N. Minakov, A. S. Chernov, V. N. Manskikh, D. S. Asyutin, N. A. Konovalov, A. G. Gabibov

**Affiliations:** Branch of Shemyakin and Ovchinnikov Institute of Bioorganic Chemistry of the Russian Academy of Sciences, Prospect Nauki 6, Pushchino, 142290, Russia; N.N. Burdenko National Scientific and Practical Center for Neurosurgery, RF Health Ministry, 4th Tverskaya-Yamskaya Str. 16, Moscow, 125047, Russia; M.V. Lomonosov Moscow State University, Leninskie Gory 1, Moscow, 119991, Russia; Shemyakin-Ovchinnikov Institute of bioorganic chemistry, GSP-7, Miklukho-Maklaya Str. 16/10, Moscow, 117997, Russia

**Keywords:** surgical simulation, laboratory rat, spinal cord injury, glial scar, axonal regeneration, cryoapplication, unilateral hemilaminectomy

## Abstract

We developed and verified an original, minimally invasive method for surgical simulation of a posttraumatic spinal cord glial scar in rats. The model is intended for use as a biological platform for testing the stimulation of regenerative processes in the central nervous system. Unification of the model enables one to achieve versatility both for implantation techniques and for the development of system-action approaches. Faced with a standard structural defect of the spinal cord, researchers will have the unique opportunity to test in vivo promising methods for spinal function recovery in the posttraumatic period. We developed anesthetic support, surgical tactics, and a set of rehabilitation measures for the chronic postoperative period. Experimental exposure effects were preliminarily assessed in vivo using a standard technique for recording the motor activity of rats in the postoperative period of spinal cord injury. Our final conclusions were drawn based on an analysis of histological sections of the rat spinal cord glial scar in three mutually perpendicular planes.

## INTRODUCTION

Spinal cord injury (SCI) is one of the main causes of disability [1], which is associated with the inevitable formation of a glial scar in the posttraumatic period, which prevents regenerative axonal growth and nerve impulses. Clinically, SCI is accompanied by a serious functional deficit, which may lead to irreversible paralysis of body areas distal to the injury. Several weeks after injury, 30% of patients develop posttraumatic syringomyelia, which reduces their neurological status [2].

The prospects for treating patients with spinal cord injuries will depend on the success of experimental studies based on the use of appropriate animal models. According to 3R principles, smaller animals are preferred for biomodeling. However, in the case of biomodeling on the spinal cord, which is associated with surgical intervention, minimization of the animal size faces obvious limitations due to the need for a sufficient amount of a simulated posttraumatic scar which allows for its use in the development of methods for spinal function recovery. The use of small rodents is considered most suitable for the modeling of SCI thanks to the common pathophysiology between the injury and clinical practice [3, 4]. Based on all these described conditions, species preference for use as an animal model was given to laboratory rats.

The most common SCI models in rats are closed SC injuries – compression-simulating impaction and contusion-simulating bruise. However, these models are difficult to reproduce, and they cannot be used to study spinal cord regeneration in structural injuries [3].

Our research team developed an approach to the modeling of SCI that uses an original cryoapplicator. The proposed innovative method for the modeling of a standard glial scar by means of cryoapplication is based on research by Vasiliev S.A. et al. on the cryodestruction of the spinal cord [5, 6], as well as on methods of nerve cryoanalgesia [7].

## EXPERIMENTAL

To optimally visualize and identify the anatomical structures and ensure sufficient space for the surgical procedure, we used large, 320–358 g, male SD rats with the SPF status. The animals were kept under standard conditions at the Animal Breeding Facility of the Branch of the Institute of Bioorganic Chemistry (BIBCh). All manipulations with the animals were approved by the Institutional Animal Care and Use Committee at BIBCh.

A total of 26 animals were used in experiments testing the use of cryotechnologies in the modeling of a structural defect of the spinal cord in rats: 14 rats were used at the stages of mastering of the experimental methodology, including development of a cryoconductor design, choice of the spinal cord cooling exposure, and access control, and 12 rats were involved at the stage of verification of the selected method for lowtemperature exposure (local cryoapplication).

**Preoperative preparation and anesthetic support**

The animals were placed in cages with clean bedding and water 24 h before surgery. Surgery was performed under general isoflurane inhalation anesthesia; premedication was not used.

**Surgical approach**

**Fig. 1:**
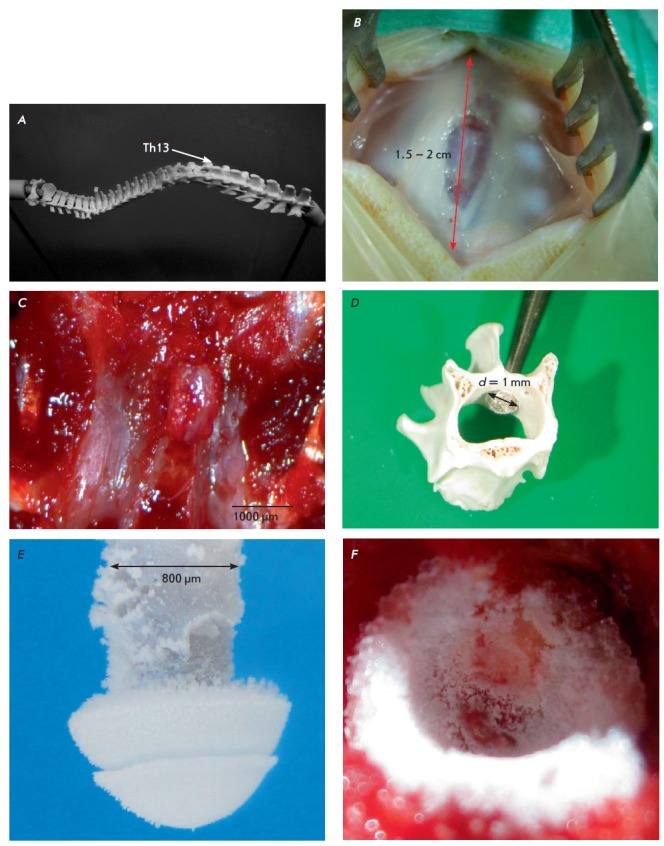
Development of the surgical approach: A – anatomical 3D-reconstruction; B – topographic landmarks, C – dissection and resection of the spinous process, D –unilateral hemilaminectomy, E – cryoprobe, F – local cooling of the spinal cord

A standard microsurgical tool kit was used to make a 2-cm median incision in the skin, subcutaneous fascia, and adipose tissue in the projection of the intersection of the vertebral column with the costal arch of the animal (Fig. 1B). The spinous process of the last thoracic vertebra T13 was identified cranial to the convergence of the posterior spinal muscle aponeuroses (musculus erector spinae) to spinous lumbar processes (Fig. 1A). The spinous process and posterior arch of the approach vertebra were skeletonized under visual control using a binocular operating microscope (Optika, Italy) (Fig. 1C). Hemostasis was provided with a thermocautery (FTS, England). The spinous process was resected to ensure sufficient operative space. Unilateral hemilaminectomy was performed using an original technique, by means of a portable dental micromotor. We used end-paramedian perforation by a 1.0-mm diamond burr at 30,000 rpm (Fig. 1D). In this case, the dura mater was not involved, which was confirmed by the absence of a CSF leak. After completion of laminectomy, the approach area was washed with physiological saline, the excess of which was synchronously removed by vacuum suction (Millipore, Germany).

**Surgical procedure**

The spinal cords in all animals in the experimental group were locally cooled by applying a cryoconductor through the dura mater (Fig. 1D, E). The conductor diameter in the area of contact with biological substrate tissue was 0.8 mm; the material was copper; the distance from the source of the cold (liquid nitrogen) in the original device was 9 cm; the exposure time for application was 1 min; the conductor temperature in the contact area was 20°C.

The animals in the control group (four rats) underwent a surgical approach to the spinal cord similar to that in the experimental rats, but without local cooling.

**Wound closure**

Tissues were closed in layers using Prolene 6/0 atraumatic nonabsorbable monofilament material (Ethicon, USA).

Single application of microporous aluminum suspension (Vetoquinol, France) was used to protect the surgical wound surface.

**Temperature mode**

During surgery, the animals were fixed on an operating table with a temperature of 38°C to compensate for the hypothermia developed upon anesthesia. Immediately after surgery and until full recovery from anesthesia, the animals were placed in a cage mounted on an electrically heated table and fed oxygen- enriched air.

**Postoperative monitoring**

The follow-up period in the experiment was 30 days. The developed surgery protocol and clinical monitoring regimen yielded a 100% survival rate of rats.

To prevent wound infection and because of the significant extent of the surgical injury and the chronic period of postoperative follow-up, preventive antibiotic therapy was performed through an intramuscular injection of Baytril (enrofloxacin, 25 mg/mL) at a dose of 10 mg/kg once a day, for 10 days.

For postoperative analgesia, the rats were treated with Norocarp (carprofen) at a dose of 10 mg/kg of live weight once a day, for 4–6 days.

For re-hydration immediately after surgery, the animals were subcutaneously injected with 5 mL of a physiological sodium chloride solution preheated to 38°C.

Throughout the chronic experiment, the operated animals were evaluated for appearance, unprovoked behavior, respiratory rate, feed and water consumption, bodily functions, nest building, response to hands, mucous membrane color, skin turgor, surgical wound condition, body weight changes, body temperature (rectal), and motor and sensitive functions of pelvic limbs and tail.

**Assessment of locomotor activity**

The effect of the experimental procedure on the locomotor activity of the rats was tested “in open field,” according to the 21-point BBB scale, which is traditionally used in the simulation of spinal cord injury [8].

**Morphological examination**

The spinal cord was isolated from the spinal canal (within the T12–L1 vertebrae) by transecting the lateral walls of the posterior vertebral arches using a burr, which ensured maximum preservation of the posterior spinal cord surface at the site of impact. The ostensible site of cold exposure was macroscopically identified by a light brown spot on the tissue, which was about 3/4 of the spinal cord diameter. Samples were fixed in a 10% formalin solution in phosphate buffer (pH 7.4) for 24 h. After fixation, the samples were dehydrated in isopropyl alcohol (Isoprep, Biovitrum), embedded in paraffin, sliced in 5-μm sections on a rotational microtome (RM2245, Leica), and stained with hematoxylin and eosin according to routine protocols [9].

To comprehensively assess the morphological features of the injury, we prepared serial sections in three mutually perpendicular planes: sagittal (to evaluate the depth and longitudinal extent of the defect), frontal (area in the contact spot plane), and segmental (transverse defect size relative to the spinal cord diameter). The sections with a maximum defect area were selected for morphological measurements in each plane. The topography of the affected spinal cord structures was determined according to the data of [10].

Sample images and morphometric procedures were produced with an Axioscope A1 microscope and a MRc 5 camera using the AxioVision 3.0 software (Carl Zeiss, Germany). The obtained data were processed using the SigmaPlot statistic (v. 13.0) software package.

## RESULTS AND DISCUSSION

**Surgical approach development**

The investigation of spinal function recovery requires the modeling of a posttraumatic glial scar – standard and minimal in volume. In contrast to the mainly mechanical contusion impacts on the spinal cord used to simulate spinal cord injury, we modeled the glial scar using an original technique of local low-temperature spinal cord injury. The idea’s prototype was the experiment of using cryotechnologies for cryoanalgesia of peripheral nerves and cryodestruction of central nervous system tissue [5, 6, 7].

To ensure the most correct interpretation of the results obtained in the model of the posttraumatic spinal cord glial scar during the development of methods for spinal function recovery, we chose a unilateral spinal cord impact which enabled the use of clinical and pathomorphological changes to the intact state as a control.

Due to the need to standardize the spinal cord approach level, we attempted original approaches, such as using the topography of the convergence of the posterior spinal muscle aponeuroses as an intraoperative landmark of the last thoracic vertebra from the side of the operational action vector (Fig. 1B) and using the dura mater as a damper for direct cold effect on the spinal cord. It should be noted that intraoperative navigation may be significantly complicated in younger animals, which are smaller in size, as well as in rats with dark pigmentation (e.g., the Dark Agouti line) because the posterior spinal muscle aponeuroses involved in the lumbar spinous processes look much less contrasting than those in large albino rats.

**Fig. 2:**
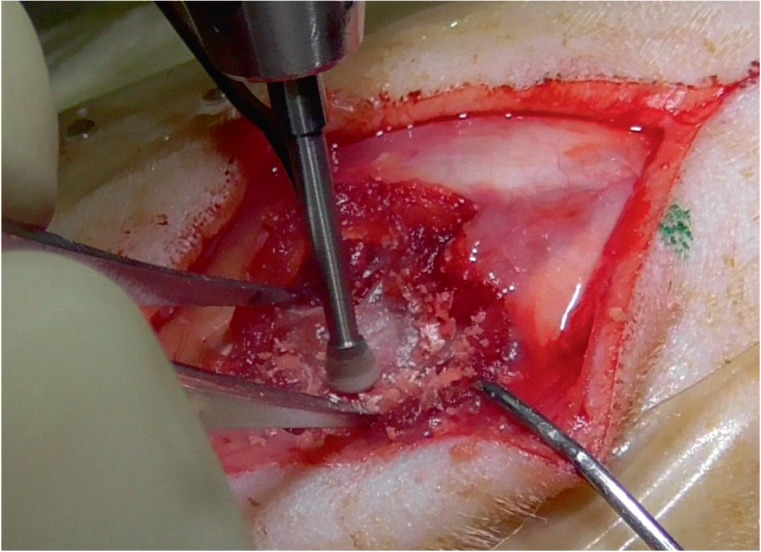
Hemilaminectomy of the T13 posterior arch in a rat, using a dental burr

Therefore, the technique for applying a cryoconductor to the spinal cord enables maximal localization of the low-temperature exposure site and minimization of nonspecific concussive organ damage during surgery. An indicator of structural preservation of the spinal cord during unilateral hemilaminectomy performed by end perforation was a lack of CSF leaks. Animals with iatrogenic durotomy were excluded from the experiment. A transosseous approach to the spinal cord was performed using a dental burr (Fig. 2).

**Clinical monitoring indicators**

According to the BBB scale [9], monoplegia was observed on the exposure side in most rats with a simulated posttraumatic scar of the spinal cord, which lasted for 21 days. The animals in the experimental group had a medium level of locomotor dysfunction (2.3 points of the BBB scale), while in the control group lacking cold exposure complete recovery of the motor activity was observed 5 days after surgery.

Current SCI models cause significant urinary system dysfunctions in rats, which is a serious drawback [11]. It is necessary to manually empty the bladder of the animals several times a day after injury to avoid bladder rupture and infectious inflammation [12, 13]. Our model had no such drawback thanks to the minimal surgical injury. After injury, the animals retained their ability to naturally empty their bladder and intestines during the entire follow-up period despite the persistent monoplegia. The ability to urinate independently and defecate is the key to life support in the chronic postoperative period; it prevents the development of distress in rats and a nonspecific injury to the spinal cord when stimulating natural movements by palpation on the walls of the intestines and bladder through the abdominal wall of the animal.

**Histological findings**

**Fig. 3:**
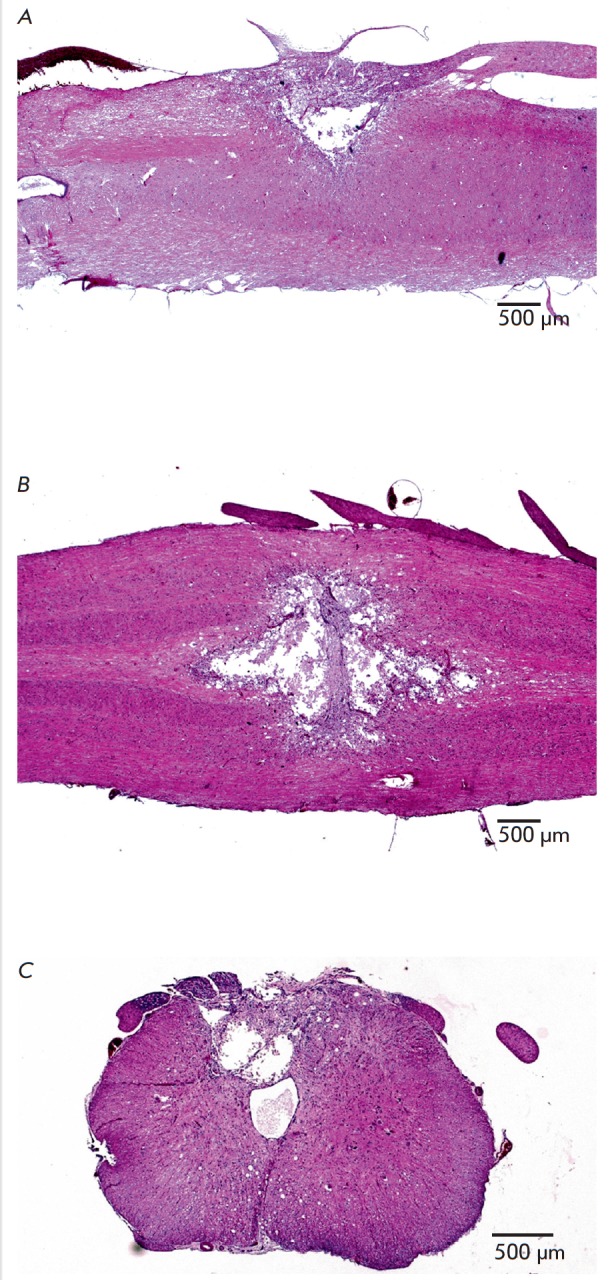
Typical histological pattern of spinal cord cryodestruction on day 30 after the proposed surgical procedure: A – sagittal section (the walls of a crater-shaped defect are depicted by arrows). B – frontal section (the structural defect cavity is clearly visible); C – segmental section (the structural defect cavity is clearly visible). H&E staining, ×25 magnification

According to the spinal cord histological data, an identical histological pattern was observed in all animals on the 30th day after cryodestruction (Fig. 3). At the defect center, there was a cavity filled with cell debris and macrophage-like cells. The cavity was partially lined with elements of a maturating gliomesodermal scar, which were more pronounced on the meningeal side and formed cords and commissures. Most of the wall, including areas adjacent to the scar tissue, was represented by fibrous and vacuolated neuropil that gradually transformed into intact nervous tissue. In the vacuolization area, there were no neurons but there were cells of unclear morphology with signs of apoptosis (karyorrhexis and karyopyknosis of nuclei). There was no acute exudative inflammation in the defect area, but there was moderate infiltration by lymphocytes and microglial elements. The total injury area was 3.6 ± 0.25 mm2 (n = 6) in the sagittal plane (Fig. 3A), 3.2 ± 0.36 mm2 (n = 4) in the frontal section (Fig. 3B), and 1.1 ± 0.1 mm2 (n = 4) in the cross section (Fig. 3C). In all cases of experimental exposure, the posterior horns of gray matter and the adjacent lateral funiculi in the tractus pyramidalis et tractus dorsolateralis area were structurally altered.

**Fig. 4:**
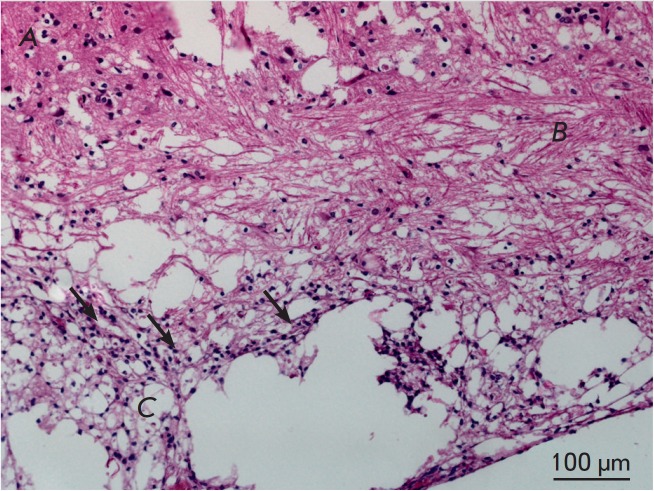
Morphology of the affected spinal cord area during hemilaminectomy (×200). A – intact tissue; B – reactive changes (neuropil rarefaction); C – necrosis area, arrows indicate cells of inflammatory infiltrate (lymphocytes or microglial elements)

It should be noted that in the control group, where only hemilaminectomy was performed, a gliomezodermal- like scar was found in the approach area (Fig. 4). This fact may be associated with technical errors inherent to the approach technique, but it is also an indication of how much the spinal cord is sensitive to any outside influence.

Further optimization of the local spinal cord cooling technology will involve improvements to the original cryoconductor design and optimization of lowtemperature exposure conditions. Detailed studies on animal models related to the improvement of surgical techniques and use of innovative laser-optical technologies and other techniques will bring practicing surgeons closer to solving the problem of functional recovery of the spinal cord in the posttraumatic period [14, 15, 16].

## CONCLUSION

This study substantiated, developed, and verified a method for the simulation of a posttraumatic spinal cord glial scar in rats. Exact adherence to the established anatomical landmarks, as well as the use of original tools and methods of local cold exposure of the spinal cord, enabled the unification of the simulated object. According to the clinical and histological data harvested, the proposed technique indicates the creation of an adequate animal model of posttraumatic spinal cord scar.

## Abbreviations

SC: spinal cord
SCI: spinal cord injury
3Rs: principles (Reduce, Refine, Replace) for performing more humane animal research
BBB scale: a scale (Basso, Beattie, Bresnahan) to evaluate locomotor recovery in rats with spinal cord injury
SPF: specified pathogen free
